# Functional Genomic Analysis of *Candida albicans* Adherence Reveals a Key Role for the Arp2/3 Complex in Cell Wall Remodelling and Biofilm Formation

**DOI:** 10.1371/journal.pgen.1006452

**Published:** 2016-11-21

**Authors:** Jason A. Lee, Nicole Robbins, Jinglin L. Xie, Troy Ketela, Leah E. Cowen

**Affiliations:** Department of Molecular Genetics, University of Toronto, Toronto, Ontario, Canada; University of California Merced, UNITED STATES

## Abstract

Fungal biofilms are complex, structured communities that can form on surfaces such as catheters and other indwelling medical devices. Biofilms are of particular concern with *Candida albicans*, one of the leading opportunistic fungal pathogens of humans. *C*. *albicans* biofilms include yeast and filamentous cells that are surrounded by an extracellular matrix, and they are intrinsically resistant to antifungal drugs such that resolving biofilm infections often requires surgery to remove the contaminated device. *C*. *albicans* biofilms form through a regulated process of adhesion to surfaces, filamentation, maturation, and ultimately dispersion. To uncover new strategies to block the initial stages of biofilm formation, we utilized a functional genomic approach to identify genes that modulate *C*. *albicans* adherence. We screened a library of 1,481 double barcoded doxycycline-repressible conditional gene expression strains covering ~25% of the *C*. *albicans* genome. We identified five genes for which transcriptional repression impaired adherence, including: *ARC18*, *PMT1*, *MNN9*, *SPT7*, and *orf19*.*831*. The most severe adherence defect was observed upon transcriptional repression of *ARC18*, which encodes a member of the Arp2/3 complex that is involved in regulation of the actin cytoskeleton and endocytosis. Depletion of components of the Arp2/3 complex not only impaired adherence, but also caused reduced biofilm formation, increased cell surface hydrophobicity, and increased exposure of cell wall chitin and β-glucans. Reduced function of the Arp2/3 complex led to impaired cell wall integrity and activation of Rho1-mediated cell wall stress responses, thereby causing cell wall remodelling and reduced adherence. Thus, we identify important functional relationships between cell wall stress responses and a novel mechanism that controls adherence and biofilm formation, thereby illuminating novel strategies to cripple a leading fungal pathogen of humans.

## Author Summary

Most microorganisms exist in surface-associated communities known as biofilms in their natural environment. These biofilms can pose a major threat to human health, as with those formed by the opportunistic fungal pathogen *Candida albicans*, which is capable of colonizing medical devices such catheters and pacemakers, thereby causing life-threatening systemic infections. The initial step in the production of a *C*. *albicans* biofilm is the adherence to a solid surface. We performed a high-throughput functional genomic screen to uncover mutants that are defective in binding to a solid surface. This enabled us to identify a novel role for the Arp2/3 complex in adherence and biofilm formation. Consistent with the importance of the Arp2/3 complex in regulating actin cytoskeleton dynamics, depletion of genes encoding Arp2/3 components caused impaired cell wall integrity. Perturbation of the Arp2/3 complex also caused increased cell surface hydrophobicity, cell wall remodelling, and hyperactivation of cell wall stress pathways mediated by the small G-protein Rho1. Thus, this study identified a previously unknown genetic relationship between the Arp2/3 complex and Rho1 in *C*. *albicans*, and uncovered novel cellular regulators that could be targeted in the development of therapeutics that block the formation of fungal biofilms.

## Introduction

In nature, the vast majority of microorganisms exist in association with surfaces in structured communities known as biofilms. Fungal biofilms are a major cause of human mortality and are highly recalcitrant to most treatments due to intrinsic drug resistance, necessitating costly surgical procedures to remove contaminated implanted devices [1,2]. A leading cause of fungal biofilm infections is *Candida albicans*, which is a commensal of the human gastrointestinal and gastrourinary tracts that is usually benign in the context of a healthy human host. However, if the immune function of the host becomes impaired or an environmental niche becomes available, it can cause serious and life-threatening infections. *Candida* ranks as the seventh most common cause of hospital-acquired infections in the United States [3], with estimated mortality rates of up to 50% despite antifungal therapy [4]. Biofilms of *C*. *albicans* contaminate and grow on medically implanted devices such as catheters, pacemakers and prostheses and are the third leading cause of intravascular catheter-related infections [2,5]. They can also colonize mucosal surfaces from which they can seed systemic infections. Thus, biofilm formation is a key virulence trait for this opportunistic pathogen and poses a significant threat to human health.

*C*. *albicans* biofilms are highly structured communities consisting of both yeast and filamentous cells surrounded by an extracellular matrix. Biofilm development occurs in four sequential stages: (i) adherence and colonization of round budding yeast cells to a surface; (ii) growth and proliferation of yeast cells to produce a basal layer of anchoring cells; (iii) growth and proliferation of filamentous cells coupled with the production of an extracellular matrix; and (iv) dispersal of yeast cells from the mature biofilm to initiate additional microbial communities [2,5]. Adherence is a critical step in the formation of *C*. *albicans* biofilms and thus defining mechanisms important for surface binding has the potential to unveil novel strategies to prevent the development of these communities.

Recent genetic and molecular studies have dramatically increased our understanding and appreciation of the complex and highly regulated process of adherence. There are a myriad of factors including cell surface structures such as pili, secreted extracellular matrix material, as well as adhesins and other cell surface proteins that coordinate the attachment of *C*. *albicans* to a surface [2,5]. Thirty transcriptional regulators have been implicated in orchestrating the genomic changes required to mediate cell-to-surface attachment [6]. This includes several transcription factors that coordinately govern the expression of 37 cell surface protein genes that are critical for adherence [6]. In addition to modulating cell surface proteins at the gene expression level, cell wall modifying proteins are implicated in surface binding through the addition or removal of chemical groups that alter the properties of cell wall proteins. For example, the mannosyltransferase Pmt1 initiates O-glycosylation of cell wall proteins in *C*. *albicans* [7], and homozygous deletion of *PMT1* impairs adherence, blocks biofilm formation, and alters cell wall composition by increasing the levels of chitin and 1,6-β-glucan-linked proteins [7,8].

In this study, we leveraged a *C*. *albicans* functional genomic library covering ~25% of the genome to identify novel regulators of biofilm adherence. The gene replacement and conditional expression (GRACE) collection consists of 1,481 double-barcoded heterozygous gene deletion mutants where the expression of the remaining wild-type allele of a gene in the diploid pathogen is governed by a doxycycline-repressible promoter [9]. We performed a high throughput pooled adherence assay with the GRACE strains to identify novel regulators of adherence. We focused our analysis on non-essential genes, and identified 15 genes for which transcriptional repression caused reduced adherence. Follow-up assays confirmed strong adherence and biofilm defects for five mutants corresponding to genes that have not been previously implicated in cell-to-surface binding, except for the previously reported role of *PMT1* [7,8]. The most severe defect in adherence was observed upon transcriptional repression of *ARC18*, which encodes a component of the Arp2/3 complex with no known role in biofilm formation or adherence. Further analysis of the molecular mechanisms orchestrating adherence led to the discovery that impaired Arp2/3 function leads to the activation of the small GTPase Rho1, culminating in a dramatic remodelling of the fungal cell wall and negatively impacting the ability of *C*. *albicans* to bind to solid surfaces. Thus, our functional genomic approach revealed a novel link between the Arp2/3 complex and Rho1 in mediating *C*. *albicans* adhesion, and suggests new strategies to block the elaboration of drug-resistant reservoirs of infection.

## Results

### Global analysis of adherence regulators

**Fig 1:**
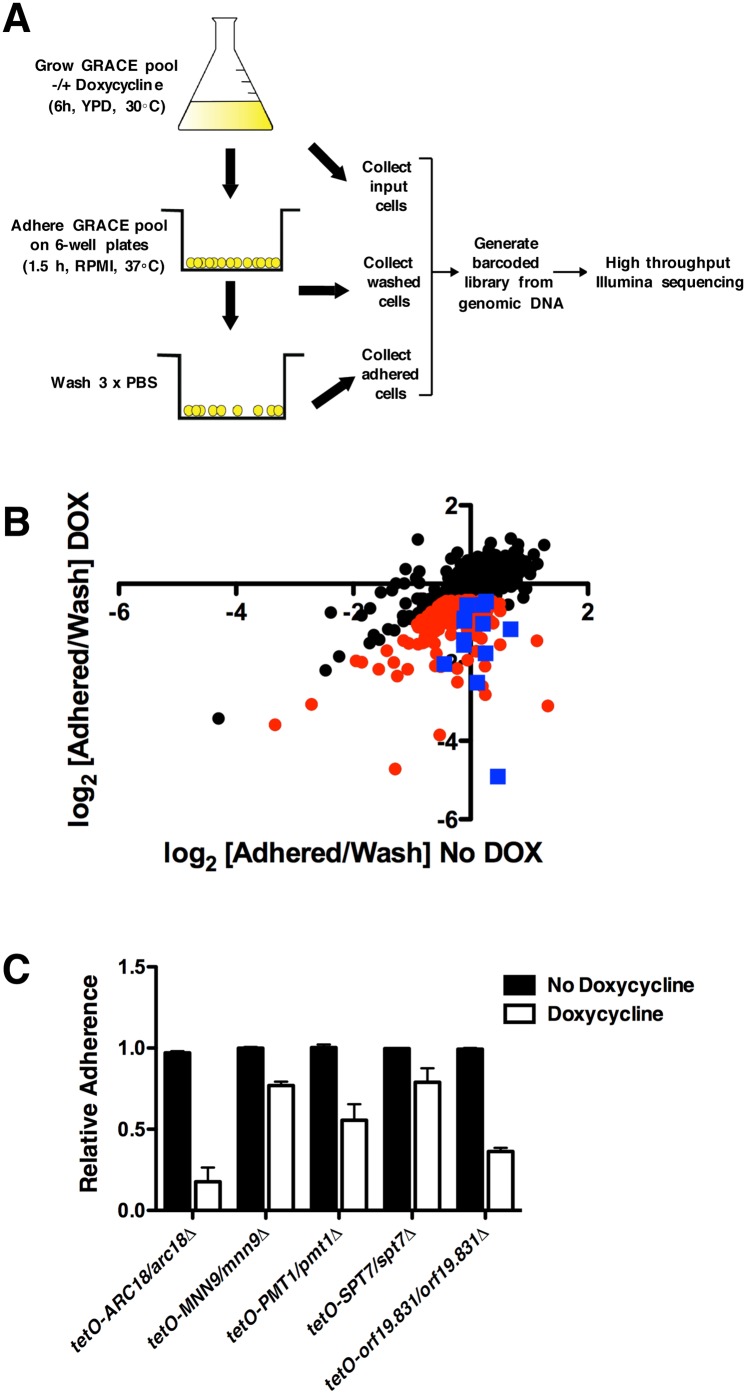
Pooled screening of the *C*. *albicans* GRACE library conditional expression strains identifies genes for which transcriptional repression causes reduced adherence. (**A**) Experimental design. Pooled strains from the GRACE library were cultured in YPD ± 0.5 μg/ml doxycycline for 6 hours. The cultures were re-suspended in RPMI and allowed to adhere to 6-well plates for one hour, at which point wells were washed three times with PBS. Cells representing input, adhered, and non-adhered (wash) fractions were collected separately. Genomic DNA was extracted from each fraction, molecular barcodes were PCR amplified, and libraries prepared for Illumina sequencing. (**B**) Scatterplot of barcode reads for the adherence screen in the absence (x-axis) and presence (y-axis) of doxycycline. Red dots represent strains with ≥4 MAD log_2_ fold difference between adhered and non-adhered (wash) normalized reads in the presence of doxycycline, with minimal effect on adherence in the absence of doxycycline. Blue squares represent strains with significant adherence defects, as defined with the red dots, and that correspond to non-essential genes. **(C)** Select strains identified in the pooled-adherence assay were grown overnight in the absence or presence of 0.5 μg/ml doxycycline. Strains were inoculated into 96-well microtiter plates in RPMI at 37°C. After one hour, wells were washed with PBS to remove non-adherent cells. The remaining adhered cells were imaged with an inverted microscope at 100x magnification. Pixel intensity of each image was quantified and normalized to the pixel intensity observed in a wild-type strain in the absence of doxycycline. Error bars represent standard deviations of five technical replicates. The experiment was performed in biological triplicate with a representative image shown. Transcriptional repression of all target genes significantly impaired the ability of the cells to adhere (*P*<0.001, ANOVA, Bonferroni's Multiple Comparison Test).

We have optimized a functional genomics platform for massively parallel analysis of fitness using next generation sequencing to quantify the relative proportion of each barcoded strain present in the GRACE collection. This approach has been successful in defining the essential gene set in *C*. *albicans* and identifying novel regulators of morphogenesis [10]. Here, we modified our platform in order to identify regulators of surface adherence. A pooled collection of 1,481 GRACE strains covering ~25% of the genome was grown in YPD in the presence or absence of 0.5 μg/ml doxycycline for 6 hours in order to repress the expression of the target genes. Cells were resuspended in RPMI and allowed to adhere to bovine serum primed 6-well plates for one hour, at which point non-adherent cells were washed away with PBS. Those cells that remained bound to the surface were collected in a fraction distinct from those cells that were efficiently washed away with PBS, and the relative abundance of each strain was assigned by sequencing the strain-specific barcodes (Fig 1A). The relative abundance of each strain was compared between the two different fractions to identify specific mutants for which transcriptional repression of the target gene impaired adherence (Fig 1B). Doxycycline-treated strains with an adherence fraction:wash fraction log_2_ ratio ≥ 4 median absolute deviations (MADs) were scored as defective in adherence (Fig 1B and S1 Table). We excluded strains from our analysis that were previously identified as essential [9] to avoid confounding effects of impaired proliferation due to essentiality.

Based on these cutoffs, we identified 15 genes for which transcriptional repression culminated in a strong defect in adherence (Fig 1B and S1 Table). For example, transcriptional repression of *PMT1* impaired adherence, providing validation that our approach is an effective strategy to identify adherence regulators [7]. We validated selected hits from our screen by performing adherence assays with each strain individually and focusing on genes that were not previously implicated in adherence. We included the *tetO-PMT1/pmt1Δ* strain in our follow-up experiments as a control. A strong and significant impairment of adherence was observed upon transcriptional repression of *ARC18*, *MNN9*, *PMT1*, *SPT7*, and *orf19*.*831* (Fig 1C, *P*<0.001, ANOVA, Bonferroni's Multiple Comparison Test). These genes are important for processes such as endocytosis (*ARC18*), transcriptional regulation (*SPT7*), and cell wall biosynthesis (*MNN9* and *PMT1*). Thus, pooled screens with the GRACE collection provide a powerful, quantitative, and high-throughput approach to identify novel *C*. *albicans* regulators of cell-to-surface adhesion.

### Mutants with adherence defects identify novel regulators of biofilm formation

**Fig 2:**
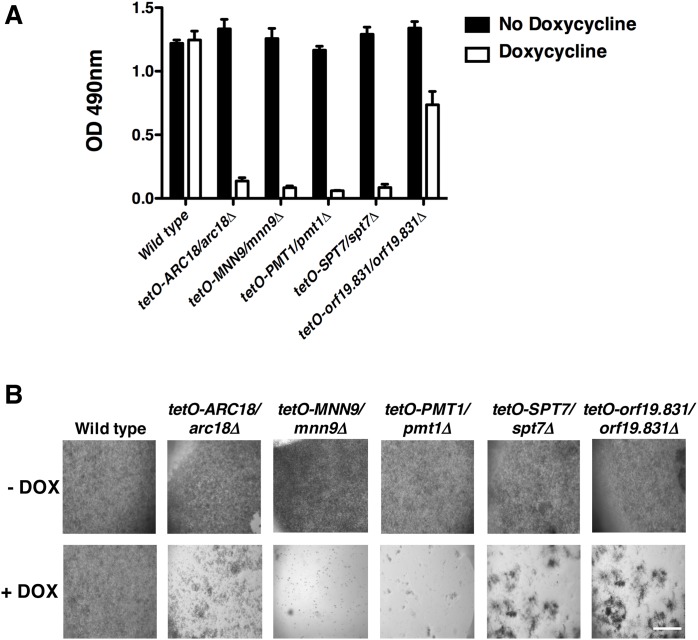
Strains with reduced adherence are impaired in biofilm formation. (**A**) Strains with adherence defects were grown overnight in the absence or presence of 0.5 μg/ml doxycycline. Biofilms were grown in 96-well microtiter plates in RPMI at 37°C for 24 hours. Metabolic activity was measured using an XTT reduction assay and quantified by measuring absorbance at 490 nm. Error bars represent standard deviations of four technical replicates. Transcriptional repression of all target genes significantly impaired the ability of the cells to form biofilms (*P*<0.001, ANOVA, Bonferroni's Multiple Comparison Test). **(B)** Representative images of biofilms formed in 96-well microtitre plates as viewed using Zeiss stereoscope. Scale bar represents 3 mm.

Central to the formation of fungal biofilms is the ability of microbial cells to adhere to substrates. Given that we identified several novel regulators of surface binding, we examined the capacity of the corresponding mutants to form biofilms. When strains were grown in the absence of doxycycline, no significant differences in biofilm metabolic activity were observed (Fig 2A, *P*>0.05, ANOVA, Bonferroni's Multiple Comparison Test). However, doxycycline-mediated transcriptional repression of the target genes significantly reduced biofilm formation for all strains (Fig 2A, *P*<0.001). Visual inspection by microscopy confirmed our metabolic assay, as strains with reduced metabolic activity showed a substantial reduction in cells that had grown on the polystyrene surface compared with the robust monolayer that was apparent in conditions where metabolic activity was unaffected (Fig 2B). To our knowledge, all of the genes that we identified as having a profound impact on biofilm formation encode novel regulators of biofilm formation, with the exception of *PMT1*. Thus, our functional genomic analysis of adherence regulators uncovered new genes critical for biofilm formation.

### Characterizing cell wall physiology in adherence mutants

**Fig 3:**
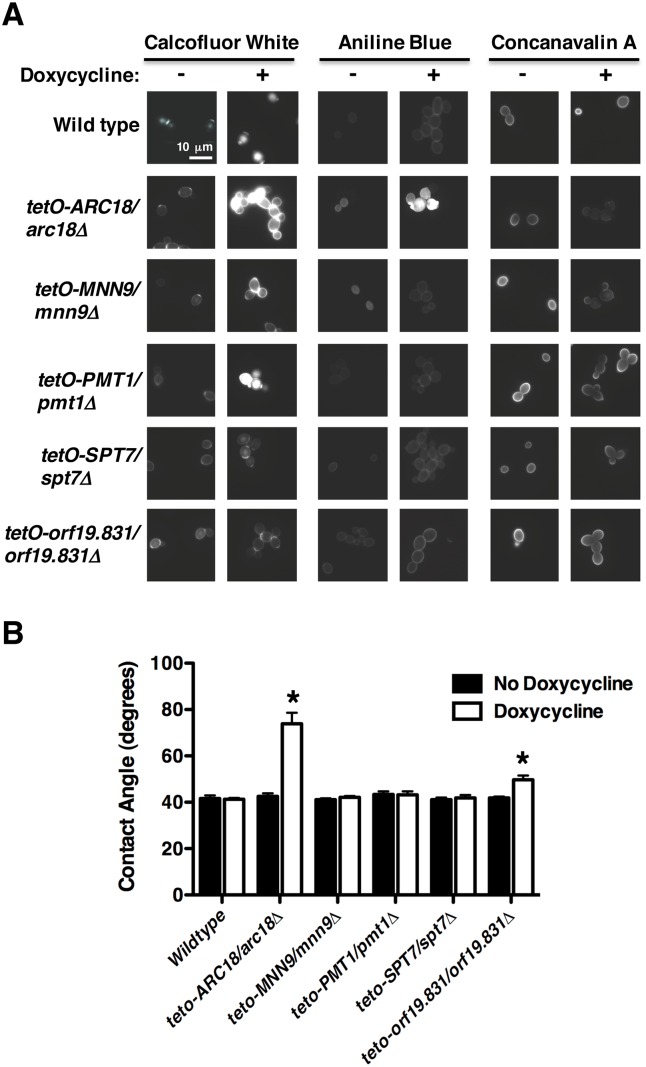
Many strains with reduced adherence have aberrant cell wall physiology. **(A)** Representative images of adherence-defective strains cultured in the absence and presence of doxycycline. Levels of cell wall components were monitored by staining with Calcofluor White (chitin), Aniline Blue (glucan), and Concanavalin A (mannans). **(B)** Cell surface hydrophobicity upon transcriptional repression of genes important for adherence. Cells were subcultured in YPD ± 0.5 μg/ml doxycycline for 20 hours, and dried on agar-glycerol medium. Contact angle measurements were calculated from the diameter of a 3 μl droplet of water on dried cell surfaces at 200 ms. Error bars represent standard deviations of nine technical measurements. Experiment was repeated in duplicate and representative image is shown. Asterisks indicate where transcriptional repression of target genes significantly altered cell surface hydrophobicity (*P*<0.001, ANOVA, Bonferroni's Multiple Comparison Test).

Given that proper cell wall composition is poised to influence adherence [6,11], we assessed whether our adhesion-defective mutants displayed gross alterations in their cell wall physiology. The diagnostic fluorescent stains Calcofluor White, Aniline Blue, and Concanavalin A were used to monitor the levels of chitin, glucan and mannan, respectively. Compared to the wild type, the *tetO-ARC18/arc18Δ* strain exhibited a marked increase in fluorescence intensity upon staining with Calcofluor White and Aniline Blue in the presence of doxycycline (Fig 3A), indicating levels of chitin and β-glucans are enhanced in response to transcriptional repression of *ARC18*. In contrast, depletion of *ARC18* caused decreased staining with Concanavalin A (Fig 3A), suggesting that lower levels of mannans were present in the cell walls. Other strains that displayed increased chitin staining when grown in doxycycline include *tetO-MNN9/mnn9Δ* and *tetO-PMT1/pmt1Δ* (Fig 3A). Interestingly, the *tetO-orf19*.*831/orf19*.*831Δ* strain did not display any changes in cell wall staining relative to wild type, suggesting that gross changes in cell wall architecture do not contribute to this mutant’s adherence defect.

To further investigate the cell wall physiology of our adherence-defective mutants, we performed a contact angle assay to measure the cell surface hydrophobicity of the adherence-defective strains (Fig 3B). A compelling body of evidence suggests that alterations in cell surface hydrophobicity due to the presence or absence of hydrophobic membrane proteins or the formation of germ tubes impacts *Candida* adhesion and biofilm formation [12–14]. Cell surface hydrophobicity can be quantified by measuring the value of the water contact angle when a droplet of water is placed on a layer of cells. If the value is over 50 then the surface in considered hydrophobic, and if the value is below 50 then the surface is considered hydrophilic [15]. Measurement of the cell surface hydrophobicity of a wild type *C*. *albicans* strain in the presence or absence of doxycycline yielded a contact angle of 40 (Fig 3B), consistent with previously published results [15]. In the absence of doxycycline, all adherence mutants showed contact angle measurements similar to wild type. However, contact angle measurements increased upon doxycycline-mediated transcriptional repression of *ARC18* or *orf19*.*831*, indicating an increase in hydrophobicity in these mutants (Fig 3B, *P*<0.001, ANOVA, Bonferroni's Multiple Comparison Test). Collectively, this work establishes cell surface physiology changes for several adherence-defective mutants, with the *tetO-ARC18/arc18Δ* strain demonstrating gross abnormalities in cell wall architecture and cell surface hydrophobicity.

### The Arp2/3 complex plays a pivotal role in adherence, biofilm formation, and cell wall physiology

**Fig 4:**
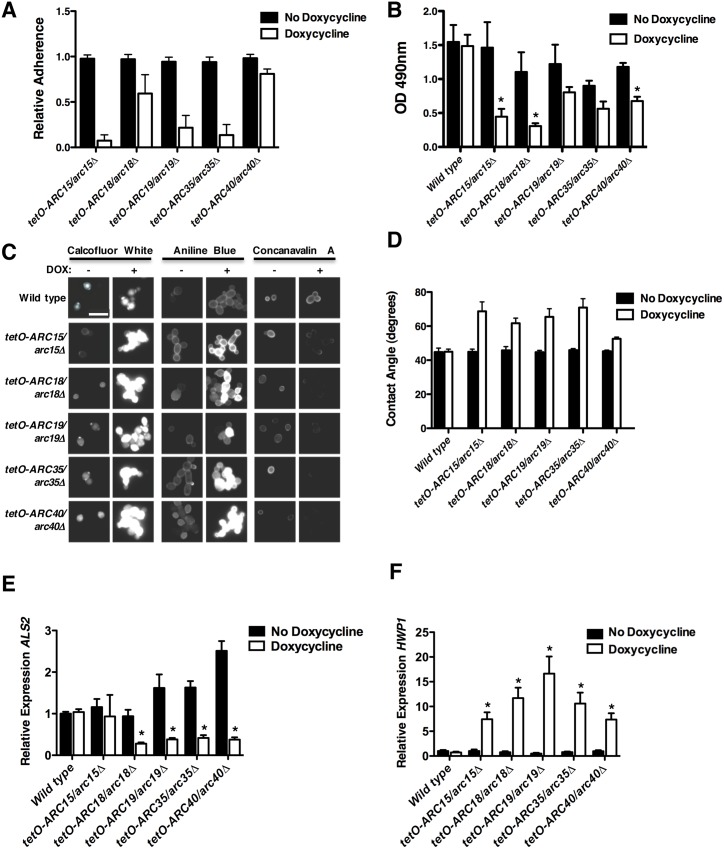
The Arp2/3 complex plays a critical role in adherence, biofilm formation and cell wall physiology. **(A)** Non-barcoded conditional expression strains for Arp2/3 complex components were grown overnight in the absence or presence of doxycycline. Adherence was measured as described in Fig 1C. Error bars represent standard deviations of five technical replicates. Transcriptional repression of all target genes significantly impaired adherence (*P*<0.001, ANOVA, Bonferroni's Multiple Comparison Test). **(B)** Transcriptional repression of Arp2/3 complex components reduces biofilm formation. Biofilms were grown and metabolic activity was measured as described in Fig 2a. Error bars represent standard deviations of three technical replicates. Asterisks indicate significantly altered biofilm formation upon transcriptional repression of target genes (*P*<0.05, ANOVA, Bonferroni's Multiple Comparison Test). **(C)** Transcriptional repression of Arp2/3 complex components alters cell wall physiology. Staining and visualization of cells was performed as described in Fig 3A. Scale bar represents 10 μm. **(D)** Transcriptional repression of Arp2/3 complex components alters cell surface hydrophobicity. Experiment was performed and analyzed as described in Fig 3B. Error bars represent standard deviations of nine technical measurements. Transcriptional repression of Arp2/3 components significantly alters cell surface hydrophobicity (*P*<0.001, ANOVA, Bonferroni's Multiple Comparison Test). **(E)** *ALS2* or **(F)** *HWP1* transcript levels were monitored after strains were sub-cultured overnight in the absence and presence of doxycycline, followed by a sub-culture and growth to log-phase in the absence and presence of doxycycline. The expression of *ALS2* and *HWP1* was normalized to a *GPD1* control, and is plotted relative to the wild-type strain in the absence of doxycycline. Error bars represent standard deviation of technical triplicates. * indicates significant differences between untreated and doxycycline treated conditions (*P*<0.01, ANOVA, Bonferroni's Multiple Comparison Test). Experiment was performed in duplicate with a representative image shown.

Next, we investigated the mechanism by which Arc18 mediates cell-to-surface binding, given that the *tetO-ARC18/arc18Δ* strain had the greatest reduction in adherence and the most profound alteration in cell wall physiology. Arc18 is a putative component of the Arp2/3 complex, which plays important roles in the regulation of the actin cytoskeleton [16]. Other members of this complex play important roles in *C*. *albicans* virulence, morphogenesis and cell wall organization [17]; however, this complex has not been previously implicated in cellular adhesion or biofilm development. To determine whether transcriptional repression of other components of the Arp2/3 complex causes similar phenotypes to those observed in the *tetO-ARC18/arc18Δ* strain, we performed equivalent adherence, biofilm, cell surface hydrophobicity and fluorescence staining assays on non-barcoded conditional expression strains for *ARC15*, *ARC19*, *ARC35*, and *ARC40*. In the absence of doxycycline, all mutants displayed phenotypes identical to wild-type strains. However, upon transcriptional repression with doxycycline (S1 Fig), Arp2/3 complex mutants showed reduced adherence (Fig 4A), reduced biofilm formation (Fig 4B), altered cell wall staining patterns (Fig 4C), and increased cell surface hydrophobicity (Fig 4D). Thus, the Arp2/3 complex modulates cell wall physiology in *C*. *albicans*, and has a profound impact on adherence and biofilm formation. In order to further probe the mechanism by which the Arp2/3 complex regulates cell-to-surface adherence, we monitored the expression of the adhesion genes *ALS2* and *HWP1* upon transcriptional repression of members of the Arp2/3 complex. Doxycycline-mediated repression of all members of the complex except *ARC15* led to significant decreases in *ALS2* transcript levels (Fig 4E, *P*<0.01, ANOVA, Bonferroni's Multiple Comparison Test). In contrast, depletion of all members of the Arp2/3 complex caused a significant increase in *HWP1* expression (*P*<0.001) (Fig 4F). Thus, impairment of the Arp2/3 complex leads to misregulated gene expression of specific adhesins.

**Fig 5:**
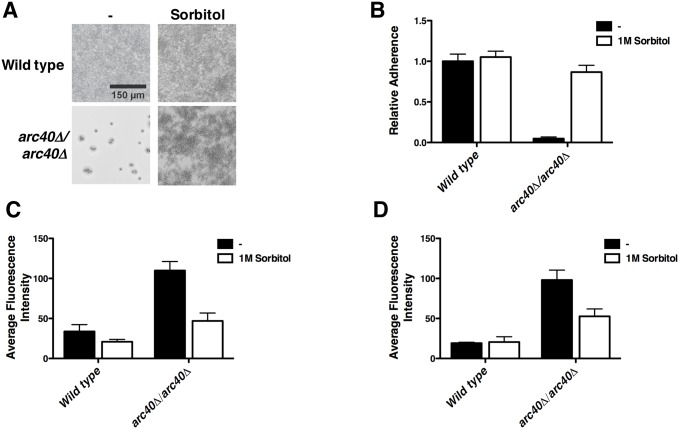
The Arp2/3 complex plays a critical role in maintaining cell wall integrity. **(A-B)** Osmotic stabilization with sorbitol rescues adherence defect of Arp2/3 complex mutants. Cells were grown in YPD ± 1M sorbitol at 30°C and adherence assay was performed as described in Fig 1C. Representative microscopy images of 96-well adherence assay are shown **(A)** and quantification of adherence is plotted in **(B)**. Error bars represent standard deviations of nine technical measurements. Sorbitol significantly restores the ability of the *arc40Δ/arc40Δ* mutant to adhere to a solid surface (*P*<0.001, ANOVA, Bonferroni's Multiple Comparison Test). **(C-D)** Treatment with sorbitol restores chitin **(C)** and glucan **(D)** content in the cell wall of an *arc40Δ/arc40Δ* mutant to wild-type levels. Microscopy was performed as described in Fig 3A. Fluorescence intensity was quantified. Error bars represent standard deviations of five technical measurements. Significant differences in fluorescence intensity upon staining with Calcofluor White and Aniline Blue were observed in an *arc40Δ/arc40Δ* mutant in the absence and presence of sorbitol (*P*<0.001, ANOVA, Bonferroni's Multiple Comparison Test).

The hyper-accumulation of chitin that was observed in the Arp2/3 complex mutants is indicative of a compensatory response to cell wall stress [18]. Hence, we hypothesized that perturbation of the Arp2/3 complex would cause defects in cell wall integrity. To test this, we constructed an *arc40Δ/Δ* mutant and assessed its ability to adhere to surfaces upon supplementation with sorbitol, an osmotic stabilizer that reduces turgor pressure and alleviates cell wall stress. Similar to the *tetO-ARC40/arc40Δ* strain, complete deletion of the *ARC40* gene led to impaired adherence relative to wild type (Fig 5A and 5B). Consistent with the adherence defect being attributable to impaired cell wall integrity, surface binding of the *arc40Δ/Δ* strain was largely restored when grown in the presence of 1M sorbitol (Fig 5A and 5B). Moreover, relative amounts of chitin and β-glucan in the *arc40Δ/Δ* fungal cell wall were partially restored to wild type levels in the presence of 1M sorbitol (Fig 5C and 5D), demonstrating that cell wall stress is the trigger for cell wall remodeling. This suggests that impaired Arp2/3 function leads to a loss of cell wall integrity, thereby causing altered cell wall physiology and impaired adherence.

### *C*. *albicans* Arp2/3 complex mutants have enhanced rates of endocytosis

**Fig 6:**
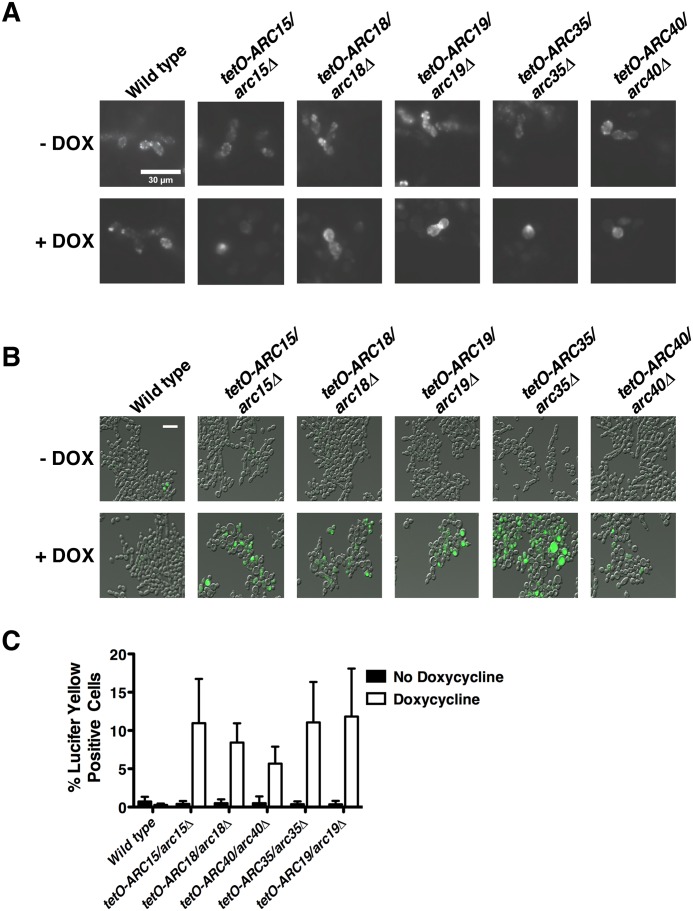
Transcriptional repression of components of the Apr2/3 complex leads to impaired actin patch formation and enhanced rates of endocytosis. **(A)** Microscopy images of Arp2/3 complex mutants grown in the absence or presence of doxycycline and then stained with Rhodamine-Phalloidin. **(B)** Microscopy images of Arp2/3 complex mutants grown in the absence or presence of doxycycline and then incubated with Lucifer Yellow. Scale bar represents 30 μm. **(C)** Quantification of endocytosis defect by counting the number of cells staining positive for Lucifer Yellow. Error bars represent standard deviations of nine technical measurements. Depletion of each component of the Arp2/3 complex significantly increases the rate of endocytosis (*P*<0.01, ANOVA, Bonferroni's Multiple Comparison Test).

It has been previously shown that *C*. *albicans* strains lacking *ARP2* or *ARP3* have dramatic actin cytoskeleton defects and delayed rates of endocytosis [17]. A common feature of cells with endocytic defects and depolarized actin patches is hyperactivity of cell wall stress pathways [19]. We assessed whether the mutants in our study also exhibited endocytic defects accompanying the activation of cell wall stress. As expected, cortical actin patches were completely absent in all Arp2/3 complex conditional expression strains upon treatment with doxycycline as visualized by staining cells with Rhodamine Phalloidin (Fig 6A and S2 Fig). Surprisingly, we did not observe impairment in endocytosis, but rather a dramatic increase in the rate of endocytosis upon transcriptional repression of *ARC15*, *ARC18*, *ARC19*, *ARC35*, or *ARC40* relative to wild type, as monitored through a Lucifer Yellow endocytosis assay (Fig 6B and 6C). These findings suggest that genetic perturbation of the Arp2/3 complex causes increased endocytosis by activating compensatory endocytic mechanisms.

### Rho1 is activated upon impairment of the Arp2/3 complex

**Fig 7:**
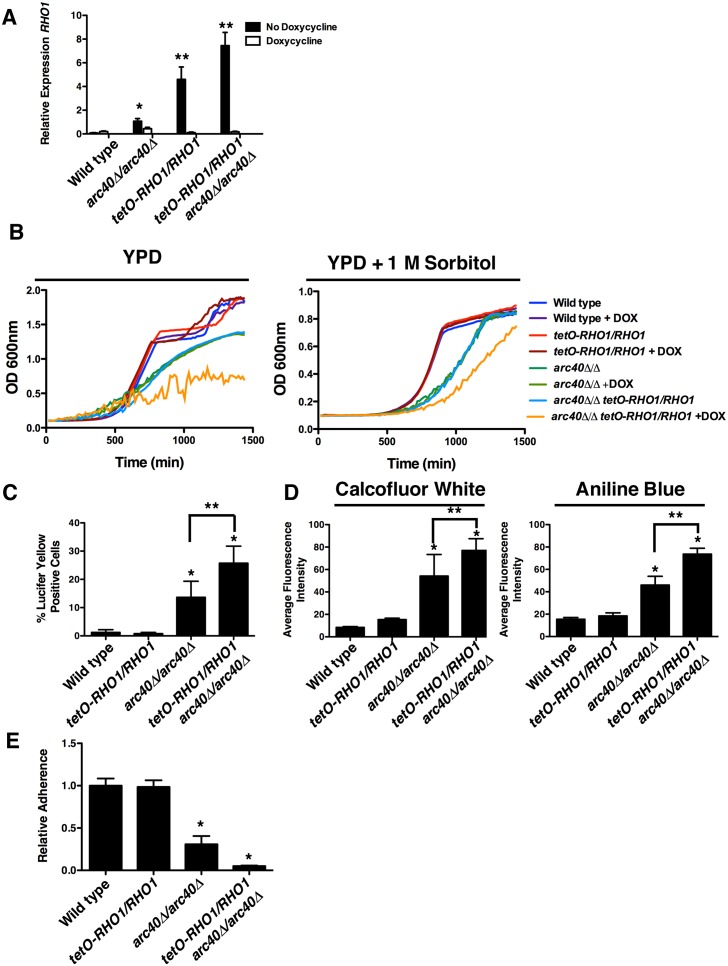
Loss of function of the Arp2/3 complex leads to hyperactivation of Rho1, altered cell wall physiology and impaired cell-to-surface adhesion. **(A)** *RHO1* transcript levels were monitored after strains were incubated for four hours in the presence and absence of doxycycline. The expression of *RHO1* was normalized to a *GPD1* control, and is plotted relative to the wild-type strain in the absence of doxycycline. Error bars represent standard deviation of technical triplicates. Experiment was performed in duplicate with a representative image shown. * indicates *P*<0.05, (ANOVA, Bonferroni's Multiple Comparison Test). ** indicates *P*<0.01. **(B)** Growth curves of strains cultured with or without doxycycline in YPD or YPD + 1M sorbitol. Growth was monitored by measuring optical density every 15 minutes. Experiment was performed in biological triplicate with representative plot shown. **(C**) Quantification of endocytosis defect by counting the number of cells staining positive for Lucifer Yellow, as described in Fig 6C. Error bars represent standard deviations of nine technical measurements. * represent significant adherence defects compared to a wild-type strain, and ** represents significant adherence defects between indicated strains (*P*<0.01, ANOVA, Bonferroni's Multiple Comparison Test). **(D)** Quantification of Calcofluor White and Aniline Blue fluorescence intensity to estimate the amount of chitin and glucan respectively in the fungal cell wall. Error bars represent standard deviation of five technical replicates. * represent significant adherence defects compared to a wild-type strain, and ** represents significant adherence defects between indicated strains (*P*<0.01, ANOVA, Bonferroni's Multiple Comparison Test). **(E)** Adherence was evaluated and measured as described in Fig 1C. Error bars represent standard deviations of five technical replicates. Asterisks represent significant adherence defects compared to a wild type strain (*P*<0.001, ANOVA, Bonferroni's Multiple Comparison Test).

In the model yeast *Saccharomyces cerevisiae*, an endocytic route has recently been identified that is independent of the canonical clathrin- and Arp2/3-dependent pathway, and instead depends upon the GTPase Rho1, the downstream formin Bni1, and the Bni1 cofactors Bud6 and Spa2 [20]. In *C*. *albicans*, Rho1-mediated endocytosis has not been characterized, but is a strong candidate for the compensatory endocytosis observed in the absence of Arp2/3 activity [17]. To test this, we constructed strains where *RHO1* expression is under the control of a doxycycline-repressible promoter in both a wild-type strain and the *arc40Δ/Δ* mutant. In the absence of doxycycline, we observed significant overexpression of *RHO1* transcript relative to wild type in both the *tetO-RHO1/RHO1* and *tetO-RHO1/RHO1 arc40Δ/Δ* strains (Fig 7A, *P*<0.01, ANOVA, Bonferroni's Multiple Comparison Test). Treatment with doxycycline resulted in transcriptional repression of *RHO1* back to wild-type levels (Fig 7A). Notably, *RHO1* transcript levels were also increased in the *arc40Δ/Δ* mutant (Fig 7A) and upon doxycycline-mediated repression of all members of the Arp2/3 complex (S3 Fig), where *RHO1* is completely under the control of its own native promoter (*P*<0.05, ANOVA, Bonferroni's Multiple Comparison Test). Thus, *RHO1* expression is influenced by the Arp2/3 complex.

Next, we assessed the impact of altered *RHO1* expression on *C*. *albicans* growth and viability. Growth of the *tetO-RHO1/RHO1* strain was comparable to wild type in the absence and presence of doxycycline, indicating that changes in *RHO1* transcript levels do not affect *C*. *albicans* growth under standard conditions (Fig 7B). Similarly, overexpression of *RHO1* in an *arc40Δ/Δ* strain also had no impact on *C*. *albicans* growth. However, transcriptional repression of *RHO1* in the *arc40Δ/Δ* mutant severely impaired growth relative to wild type (Fig 7B). This growth defect was partially rescued upon osmotic stabilization with sorbitol (Fig 7B), supporting a model that elevated levels of Rho1 are required for survival under conditions of cell wall stress.

Next, we assessed whether endocytosis mediated through Rho1 was the compensatory pathway responsible for the elevated rates of endocytosis in an Arp2/3 complex mutant. Overexpression of *RHO1* alone in the *tetO-RHO1/RHO1* strain was insufficient to cause elevated rates of endocytosis (Fig 7C). However, overexpression of *RHO1* in the *tetO-RHO1/RHO1 arc40Δ/arc40Δ* background increased rates of endocytosis to an even greater extent than that observed in the *arc40Δ/arc40Δ* mutant (Fig 7C). Similarly, overexpression of *RHO1* on its own had no observable impact on chitin or β-glucan content in the cell wall, nor did it impair cell-to-surface adherence relative to a wild-type strain (Fig 7D and 7E). However, overexpression of *RHO1* in the *arc40Δ/Δ* background caused increased chitin and β-glucan deposition (Fig 7D) and exacerbated the adherence defect of the *arc40Δ/Δ* mutant (Fig 7E). Collectively, these data support a model in which elevated levels of Rho1 alone are not sufficient to induce the signaling required for endocytosis or cell wall remodeling. However, defects in cell wall integrity induced by perturbation of Arp2/3 complex function activate an elevated pool of Rho1, thereby exacerbating effects on endocytosis, cell wall remodeling and adherence.

**Fig 8:**
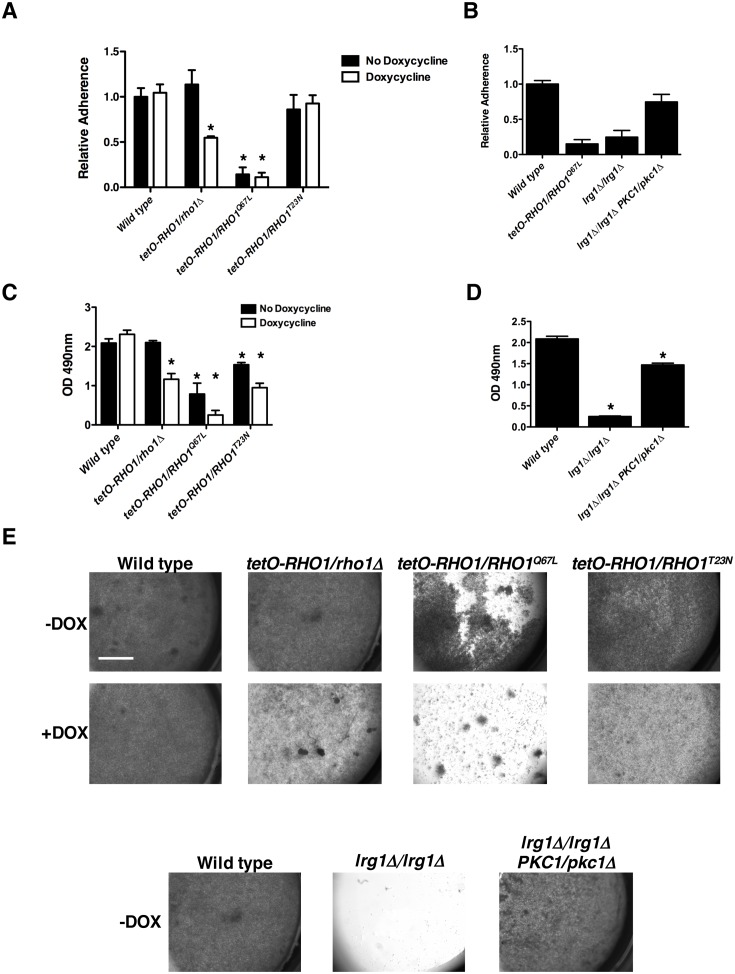
Hyperactivation of Rho1 leads to reduced adherence and biofilm formation through activation of Pkc1. **(A)** Adherence was evaluated and measured as described in Fig 1C. Error bars represent standard deviations of five technical replicates. Asterisks represent significant adherence defects compared to a wild-type strain (*P*<0.001, ANOVA, Bonferroni's Multiple Comparison Test). **(B)** Adherence was evaluated and measured as described in Fig 1C. The *tetO-RHO1/RHO1*^*Q67L*^ strain was grown in the presence of 0.5 μg/mL doxycycline. Significant differences were observed in adherence of all strains relative to both the wild-type strain and the *lrg1Δ/ lrg1Δ PKC1/pkc1Δ* strain (*P*<0.001, ANOVA, Bonferroni's Multiple Comparison Test). **(C-D)** Strains were grown overnight in the absence or presence of 0.5 μg/ml doxycycline. Biofilms were grown in 96-well microtiter plates in RPMI at 37°C for 24 hours. Metabolic activity was measured using an XTT reduction assay and quantified by measuring absorbance at 490 nm. Error bars represent standard deviations of four technical replicates. Asterisks indicate significant differences in biofilm metabolic activity relative to wild type (*P*<0.001, ANOVA, Bonferroni's Multiple Comparison Test). **(E)** Representative images of biofilms formed in 96-well microtiter plates as viewed using Zeiss stereoscope. Scale bar represents 1 mm.

To test this hypothesis, we engineered strains with constitutively active GTP-bound Rho1 (Q67L) and dominant negative GDP-bound Rho1 (T23N), where the remaining wild-type allele of *RHO1* was under the control of a doxycycline-repressible promoter. The constitutively active GTP-bound *tetO-RHO1/RHO1*^*Q67L*^ strain had significantly reduced adherence when grown in the presence or absence of doxycycline (Fig 8A), similar to what was observed in the *arc40Δ/arc40Δ tetO-RHO1/RHO1* strain (Fig 7E). However, the constitutively GDP-bound inactive *tetO-RHO1/RHO1*^*T23N*^ strain was able to adequately bind to surfaces in the presence or absence of doxycycline (Fig 8A). To determine if Rho1 signals through its known downstream effector Pkc1 to mediate adhesion [21], we deleted one allele of *PKC1* in both a wild-type strain and an *lrg1Δ/lrg1Δ* mutant. In *S*. *cerevisiae* and *C*. *albicans*, Lrg1 is a Rho1 GAP that stimulates Rho1 GTPase activity and converts Rho1 to its inactive, GDP-bound form [21,22]. Thus, homozygous deletion of *LRG1* should lock *RHO1* in a constitutively active state. We observed that deletion of *LRG1* led to reduced adherence to a similar degree as our *tetO-RHO1/RHO1*^*Q67L*^ strain in the presence of doxycycline (Fig 8B). Strikingly, deletion of only one allele of *PKC1* was sufficient to restore adherence to near wild-type levels (Fig 8B). This confirms that hyperactive Rho1 alone is sufficient to cause a major reduction in adherence through activation of Pkc1. Finally, to assess whether the defects in adherence corresponded to defects in biofilm formation we monitored biofilm formation of our mutants through quantification of metabolic activity (Fig 8C). The constitutively active GTP-bound *tetO-RHO1/RHO1*^*Q67L*^ strain had significantly reduced biofilm formation in the presence or absence of doxycycline (*P*>0.001, ANOVA, Bonferroni's Multiple Comparison Test), confirming what we observed in our adherence assay. Similarly, homozygous deletion of *LRG1* impaired biofilm formation and deletion of only one allele of *PKC1* was sufficient to restore biofilm formation to near wild-type levels (Fig 8D). Visual inspection by microscopy confirmed our metabolic assay, as strains with reduced metabolic activity showed a substantial reduction in cells that had grown on the polystyrene surface compared with the robust monolayer that was apparent in conditions where metabolic activity was unaffected (Fig 8E). These findings describe a novel functional relationship between the Arp2/3 complex and Rho1 that is important for both endocytosis and cell wall remodeling, and highlight a novel role for Rho1 in *C*. *albicans* adherence and biofilm formation.

## Discussion

**Fig 9:**
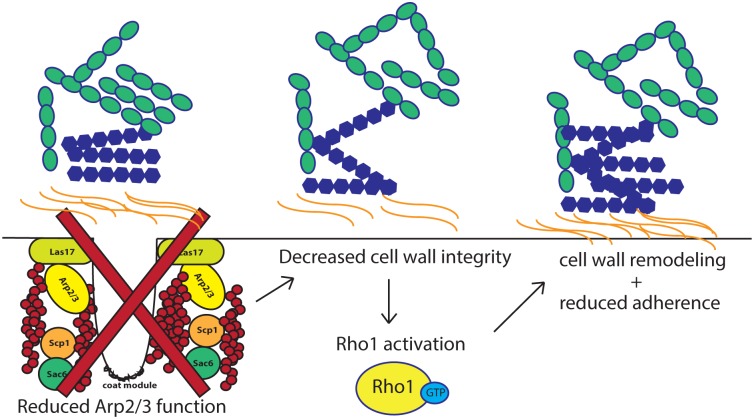
A schematic diagram depicting the mechanism by which the Arp2/3 complex governs adherence. Compromised Arp2/3 function leads to impaired cell wall integrity which leads to increased expression and activation of the small GTPase *RHO1*. Activated Rho1 orchestrates a dramatic remodeling of the fungal cell wall, causing elevated levels of chitin (orange waves) and β-glucan (blue hexagons), consequently impairing the ability of *C*. *albicans* to adhere to a surface.

The ability of *C*. *albicans* to adhere to a substrate is the initial stage of biofilm development, and a critical step for the establishment of this major class of device-associated infections. Here we describe the use of a massively parallel pooled screening platform to identify novel regulators of cell-to-surface adherence in *C*. *albicans*. This analysis expands previously identified adherence regulators to include components of the Arp2/3 complex, a highly conserved actin nucleation center required for the motility and integrity of actin patches. Compromised Arp2/3 function led to impaired cell wall integrity and hyper-accumulation of chitin as a compensatory mechanism that is deployed in response to cell wall stress. This led to increased expression of the small GTPase *RHO1*, a dramatic remodeling of the fungal cell wall, and an impaired ability of *C*. *albicans* to adhere to a surface (Fig 9). We also observed a dramatic increase in the rate of endocytosis in Arp2/3 complex mutants and implicate signaling through the small GTPase Rho1 as the compensatory pathway responsible for this phenotype. Finally, we unveil a novel role for activated Rho1 in abrogating *C*. *albicans* adhesion, and uncover a previously unidentified genetic relationship between Rho1 and the Arp2/3 complex.

*C*. *albicans* adhesion is controlled via a complex regulatory network that connects 11 adherence regulators, the zinc-responsive regulator Zap1, and 48 cell surface proteins [6]. The fact that these proteins, often referred to as **C**ell **S**urface **T**argets of **A**dherence **R**egulators (CSTARs), cover ~25% of all cell surface proteins in *C*. *albicans* and that there is such a high level of redundancy in the pathways regulating the expression of CSTARs, suggest the process of adhesion is critical for the biology of *C*. *albicans* in its natural environment. Other work that utilized a library of 531 *C*. *albicans* conditional overexpression strains, similarly described an important role for glycosylphosphatidylinositol (GPI)-modified cell surface proteins in multi-species fungal biofilm formation [11]. Despite the multiple studies that have characterized *C*. *albicans* adhesion and biofilm regulators using large-scale genomic libraries, our functional genomic pooled adherence assay was able to identify several novel regulators of adhesion and biofilm formation, including a role for the entire Arp2/3 complex. The GRACE collection encompasses only ~25% of the genome, thus, expansion of *C*. *albicans* mutant collections to genome scale has the potential to enable discovery of more complex genetic circuitry governing adhesion, biofilm formation, and cell surface physiology.

In *S*. *cerevisiae*, the Arp2/3 complex is required for the motility and integrity of cortical actin patches, and for actin-dependent processes such as endocytosis and organelle inheritance [23]. In *S*. *cerevisiae*, Rho1-mediated endocytosis has been described for both clathirin-dependent and claththrin-independent pathways [20,24]. Although previous work established that the Arp2/3 complex is not essential for endocytosis in *C*. *albicans* [17], the compensatory pathway that acts in the absence of Arp2/3 complex function remained enigmatic. Our results suggest that endocytosis through Rho1 signaling is the elusive pathway through which endocytosis occurs in the absence of Arp2/3 function in *C*. *albicans*. Genetic impairment of the Arp2/3 pathway leads to a disruption in cell wall integrity and consequently an increased expression of *RHO1*. Further, rates of endocytosis were only increased upon *RHO1* overexpression in a strain where the Arp2/3 complex was not functional. A key unresolved issue in both *S*. *cerevisiae* and *C*. *albicans* is how this alternate endocytic pathway contributes to endocytosis in cells under different conditions. It has been proposed that the Rho1 pathway might be activated by stresses such as osmotic stress and cell wall or plasma membrane damage, in order to aid rapid internalization of damaged components [20]. Our findings support this model and provide the first example of Rho1 modulating endocytosis in *C*. *albicans*.

Our study establishes that altering relative proportions of important cell wall polysaccharides can impair the ability of *C*. *albicans* to adhere to a solid surface. Tight regulation of polysaccharide levels is similarly crucial for other aspects of biofilm development. Interference with synthesis or export of any one of α-mannan, β-1,6 glucan, or β-1,3 glucan alters matrix concentrations of each of the other polysaccharides and blocks matrix sequestration of antifungal compounds [25]. The fact that in response to cell wall stress, *C*. *albicans* activates a compensatory mechanism with increased relative amounts of chitin, similar to the compensatory response observed in response to echinocandins [26], suggests a potential therapeutic strategy to block the formation of biofilms. Conceivably, medical devices coated with compounds targeting cell wall integrity should not only compromise growth of *C*. *albicans* in the planktonic state, but should also impede the ability of the fungus to adhere to medical devices. The feasibility of this idea is corroborated by a study in which caspofungin immobilization onto biomaterial surfaces was able to specifically block fungal attachment, while having limited impact on the ability of mammalian cells to attach and spread [27]. Similarly, high-throughput screens searching for adherence inhibitors unveiled chemical matter that not only blocks adherence, but also *C*. *albicans* biofilm formation and virulence [28]. Future studies to explore the *in vivo* relevance of this approach could leverage fungal-selective inhibitors of cell wall integrity signaling [29]. Localized delivery of therapeutics that block biofilm formation has the potential to prevent the establishment of highly drug-resistant communities and reduce evolutionary pressures for the emergence of drug resistance that are associated with systemic drug delivery.

## Materials and Methods

### Strains and culturing conditions

*C*. *albicans* strains were archived in 25% glycerol at -80°C. Strains were routinely maintained and grown in YPD liquid medium (1% yeast extract, 2% bactopeptone, 2% glucose) at 30°C or RPMI (Gibco) buffered with HEPES or MOPS as indicated. Strains were constructed according to standard protocols. Strain construction is described in S1 Text and strains used in this study are listed in S2 Table.

### Plasmid construction

Cloning procedures were performed following standard protocols. Plasmid construction is described in S1 Text and plasmids used in this study are listed in S3 Table. The absence of nonsense mutations on the plasmid was verified by sequencing. Primers used in this study are listed in S4 Table.

### Pooled adherence assay

A frozen stock of pooled strains from the GRACE library was thawed and diluted to an OD_600_ of 0.05 in YPD. The culture of pooled cells was grown for two hours at 30°C with shaking. After the pre-growth period, the culture of pooled cells was split into two flasks, where doxycycline (631311, BD Biosciences) was added to one of the cultures to a final concentration of 0.5 μg/ml to deplete expression of the target genes. The pooled cell cultures were grown for an additional six hours at 30°C with shaking. At this point, cells were washed with PBS and re-suspended in RPMI at an OD_600_ of 0.5 in the presence or absence of 0.5 μg/ml doxycycline. Six ml of culture suspension was added to each well of 6-well plates that had been treated with bovine serum (16190; Gibco) prior to inoculation. The plates were incubated at 37°C for 1.5 hours to allow adhesion. Next, each well was washed with three rounds of 15 ml PBS. Cell pellets were collected for sequencing analysis at different steps of the assay: (1) input cells in the RPMI cell suspension prior to the adherence step; (2) washed cells dislodged from wells during the wash step; and (3) adhered cells scraped from wells after the wash step. Genomic DNA was prepared as outlined below. Details of sequencing analysis are provided in S1 Text.

### Genomic DNA extraction

Cell pellets were resuspended in 200 μl extraction buffer (2% Triton X, 1 mM EDTA, 1% SDS, 100 mM NaCl, and 100 mM TRIS pH 8.0). To extract cellular contents, cells suspended in lysis buffer were mechanically disrupted by adding 200 μl acid-washed glass beads and bead beating for three minutes in the presence of 200 μl phenol chloroform. The aqueous layer was further purified with the addition of 200 μl of chloroform. To degrade RNA, the aqueous layer from the previous step was incubated with RNAse for 15 min at 37°C. The DNA was precipitated with 70% ethanol and 0.3 M sodium acetate at -80°C. Amplification by PCR was performed using 78 ng of genomic DNA for GRACE pool samples with the Takara Ex-Taq enzyme (Clontech #RR001). Details of thermocycling conditions are described in S1 Text.

### High throughput sequencing library preparation and data analysis

Separate UPTAG and DNTAG multiplexed pools were formed by combining equal amounts of PCR product from each sample. UPTAG and DNTAG DNA pools were electrophoresed on a 5% 1 × TBE polyacrylamide gel and recovered by eluting DNA from shredded gel slices in 10 mM Tris-HCl pH 8.0. Equal quantities of UPTAG and DNTAG pools were combined to form a library, which was sequenced on an Illumina Hi-Seq 2500 instrument (single-end flow cell) using specific primers to sequence and index the UPTAGs and DNTAGs.

Barcode sequence reads were mapped to an artificial genome containing known UPTAG and DNTAG sequences via Bowtie v1. Read frequency for the UPTAG and DNTAG of each strain were compiled for each indexed sample. Quality control procedures are described in S1 Text. Relative strain abundance in a fraction was calculated by averaging the log_2_ read counts for the UPTAG and DNTAG. Strains were considered significantly altered in adherence if the log_2_ adhered: wash ratio in doxycycline treated condition was ≥4 Median Absolute Deviations (MAD). To look for doxycycline-specific effects, we omitted strains with log_2_ adhered: wash ratios between doxycycline treated versus no doxycycline control within 1.5 fold of each other. Finally, strains with an essentiality score ≥ 4, based on essentiality score data by Roemer et al. [9] were omitted from further analysis.

### Adherence assay

Strains were subcultured in YPD at 30°C for 18 hours from an overnight culture in the presence or absence of 0.5 μg/ml doxycycline. The subcultured cells were re-suspended in RPMI at an OD_600_ of 0.5 and100 μl of cell suspension was added to each well of a 96-well plates primed with bovine serum. The plates were incubated at 37°C for 1 hour. Each well was washed twice with 100 μl of PBS. The remaining adhered cells were imaged with an Zeiss Imager M1 inverted microscope and AxioCam Mrm with AxioVision 4.7 software at 10x magnification. All images were processed using Open CV (cv2) with Python 2.7.11 or using Image J. The image histogram is a distribution of an image’s pixel intensity from 0 to 255. To obtain the background pixel intensity, the maximum pixel intensity of an empty portion of the image was used. Using the background pixel intensity as a cutoff, the fluorescence intensity was calculated by taking the average of the image histogram to accurately quantify cellular adherence within each well. Nine separate images were quantified for each experiment. Experiments were performed in biological triplicate.

### Biofilm assay

Biofilm assays were performed as described previously [30]. In brief, strains were subcultured in YPD at 30°C for 18 hours from an overnight culture in the presence or absence of doxycycline, as indicated. Subsequently, cultures were resuspended in RPMI medium buffered with HEPES or MOPS, in the presence or absence of doxycycline to an OD_600_ of 0.5. An aliquot of 100 μl was added to each well of a 96-well flat-bottom plate primed with bovine serum, followed by incubation at 37°C. After 90 minutes, the wells were gently washed twice with phosphate-buffered saline (PBS) to remove non-adherent cells, and fresh RPMI was added with or without a doxycycline. After 24 hours, non-adherent cells were washed away with PBS and biofilm cell metabolic activity was measured using the XTT reduction. Briefly, 90 μl of XTT (X4251, Sigma) at 0.5 mg/ml and 10 μl phenazine methosulfate (P9625, Sigma) at 320 μg/ml were added to each well, followed by incubation at 37°C for 15 minutes. Absorbance of the supernatant transferred to a fresh plate was measured at 490 nm using an automated plate reader, and experiments were carried out in a minimum of 5 replicates for each strain.

### Contact angle cell surface hydrophobicity assay

The contact angle cell surface hydrophobicity assay was performed as described previously [15]. In short, cells were subcultured in presence or absence of doxycycline for 18 hours from an overnight. Then, the cells were washed with ddH_2_O, and resuspended in ddH_2_O to an OD_600_ of 20. Next, cells were dried for 24–72 hours on 10% glycerol 2% agar medium in 6-well plates. To measure the contact angle, a 3 μl drop of water was added on top of the dried layer of cells. Time series set of images was taken using a stereoscope at 1x magnification. The diameter of the droplet was measured at approximately 200 ms, and the contact angle was calculated using ADSA-D.

### Fluorescent cell wall staining

Strains were subcultured in YPD at 30°C for 18 hours from an overnight culture in the presence or absence of doxycycline. Then, the cells were then washed with ddH_2_O, and resuspended in ddH_2_O to an OD_600_ of 1.0 in the presence of fluorescent dyes at the following concentrations: Aniline Blue (Sigma, 0.05% (w/v), Calcofluor White (Sigma, 1 μg/ml), Concanavalin A (Sigma, 0.1 μg/ml). The cells were imaged with Zeiss Imager M1 upright microscope and AxioCam Mrm with AxioVision 4.7 software at 40x magnification with constant exposure for each respective dye.

To quantify fluorescence, image histogram data of fluorescent microscopy was obtained for the images using ImageJ. The image histogram is a distribution of an image’s pixel intensity from 0 to 255. To obtain the background pixel intensity, the maximum pixel intensity of an empty portion of the image was used. Using the background pixel intensity as a cutoff, the fluorescence intensity was calculated by taking the average of the image histogram.

### Rhodamine phalloidin actin staining

Strains were subcultured in YPD at 30°C for 18 hours from an overnight culture in the presence or absence of doxycycline. The cells were subcultured again for 4 hours in 8.5 ml of YPD (+/- doxycycline) at 30°C shaking. The cells were fixed with formaldehyde (4% v/v). For the actin staining, the cells were washed with PBS and incubated overnight in 0.66 μM Rhodamine Phalloidin (Biotium, Hayward, CA) solution in PBS at 4°C. To image the actin, the cells were washed twice with PBS and imaged with Zeiss Imager M1 upright microscope using the the DsRed filter and mages were captured using AxioCam Mrm with AxioVision 4.7 software.

### Lucifer yellow endocytosis assay

Strains were subcultured in YPD at 30°C for 18 hours from an overnight culture in the presence or absence of doxycycline. The cells subcultured again for 3 hours in YPD (+/- doxycycline) at 30°C with shaking. Next, a 0.7 OD unit of each culture was resuspended in 100 μl of 1 mg/ml Lucifer Yellow (Invitrogen, #L682) YPD solution. To ensure proper shaking, the cells were incubated horizontally in the 30°C shaker for 1 hour. Following the incubation period, the cells were washed three times with 1 ml PBS and imaged with a Zeiss Imager M1 upright microscope and AxioCam Mrm with AxioVision 4.7 software.

### Growth curves

For growth curves, cells were grown overnight in YPD without or with doxycycline. Cultures were then diluted to an OD_600_ of 0.0625 with or without doxycycline in YPD or YPD + 1M Sorbitol in 96-well plates, and grown at 30°C with continuous shaking (TECAN GENios). OD_595_ was measured every 15 min. Data were analyzed in GraphPad Prism 4.0.

### RT-PCR

*C*. *albicans* strains were grown overnight in YPD, diluted to OD_600_ of 0.1 in the absence or presence of 0.5 μg/ml doxycycline, and grown for an additional 4 hr in duplicate. RNA was isolated and RT-PCR performed as described [31]. In brief, cultures were harvested by centrifugation at 3000 rpm for 5 min. The pellet was flash-frozen and stored at -80°C overnight. RNA was isolated using the QIAGEN RNeasy kit and cDNA was generated using the AffinityScript cDNA synthesis kit (Stratagene). qRT-PCR was carried out using the Fast SYBR Green Master Mix (Thermo Fisher Scientific) in 384-well plate with the following cycle conditions: 95°C for 10 min, repeat 95°C for 10 sec, 60°C for 30 sec for 40 cycles. Transcripts were monitored using primers listed in S4 Table. The melt curve was completed with the following cycle conditions: 95°C for 10 sec and 65°C for 5 seconds with an increase of 0.5°C per cycle up to 95°C. All reactions were done in triplicate. Data were analyzed in the Bio-Rad CFX manager 3.1. See S4 Table for primers used.

## Supporting Information

S1 Table GRACE pooled adherence analysis. A list of the mutants in the GRACE library that were screened for adherence capabilities in the absence and presence of doxycycline (sheet1) and those mutants that were found to exhibit defects in adherence but be non-essential (sheet 2).
(XLSX)

S2 Table Strains used in this study. (DOCX)

S3 Table Plasmids used in this study. (DOCX)

S4 Table Primers used in this study. (XLSX)

S1 Text Supplemental Methods and Supplemental References. (DOCX)

S1 Fig Doxycycline-mediated transcriptional repression of Arp2/3 complex genes in the corresponding GRACE strains. Transcriptional repression of members of the Arp2/3 complex was achieved with 0.5 μg/ml doxycycline. Overnights were subcultured for 24 hrs in the presence or absence of doxycycline. The strains were subcultured again in the same conditions for 4 hrs. cDNA was prepared from total RNA for qRT-PCR. The transcript level of *ARC15*, *ARC18*, *ARC19*, *ARC35* and *ARC40* was monitored and normalized to *GPD1*. Data are plotted as means ± SD for triplicate samples and are representative of two independent experiments.
(TIF)

S2 Fig Transcriptional repression of components of the Apr2/3 complex leads to impaired actin patch formation. Microscopy images of Arp2/3 complex mutants grown in the absence or presence of doxycycline, and then stained with Rhodamine-Phalloidin. Scale bar represents 10 μm.
(TIF)

S3 Fig Transcriptional repression of members of the Arp2/3 complex leads to increased *RHO1* expression. Overnight cultures were subcultured for 24 hrs in the presence or absence of 0.5 μg/ml doxycycline. The strains were subcultured again in the same conditions for 4 hrs. cDNA was prepared from total RNA for qRT-PCR. The transcript level of *RHO1* was monitored and normalized to *GPD1*. Data are plotted as means ± SD for triplicate samples and are representative of two independent experiments. Asterisks indicate significant differences in *RHO1* transcript level upon treatment with doxycycline (*P*<0.05, ANOVA, Bonferroni's Multiple Comparison Test).
(TIF)
