# Supplementary material for: Functional Genomic Analysis of Candida albicans Adherence Reveals a Key Role for the Arp2/3 Complex in Cell Wall Remodelling and Biofilm Formation
Source: PLoS Genet. 2016 Nov 21;12(11):e1006452. doi: 10.1371/journal.pgen.1006452 (PMC5147769; doi:10.1371/journal.pgen.1006452)
Supplement: S2 Table — (DOCX) [file pgen.1006452.s002.docx]

Table S2. Strains used in this study.

| Strain name | Strain number | Genotype | Reference |
| --- | --- | --- | --- |
| SN95 | CaLC239 | *arg4/arg4 his1/his1 URA3/ura3::imm434 IRO1/iro1::imm434* | [1] |
| CaSS1 | CaLC3365 | See Reference | [2] |
| *tetO-RHO1/RHO1* | CaLC4403 | *his3::hisG/his3::hisG leu2::tetRGAL4AD-URA3/LEU2 tetO-RHO1/RHO1* | This study. |
| *tetO-SPT7/spt7/Δ* |  | See Reference | [2] |
| *tetO-orf19.831/orf19.831/Δ* |  | See Reference | [2] |
| *tetO-ARC18/arc18Δ* |  | See Reference | [2] |
| *tetO-PMT1/pmt1Δ* |  | See Reference | [2] |
| *tetO-MNN9/mnn9Δ* |  | See Reference | [2] |
| *tetO-ARC15/arc15Δ* |  | See Reference | [2] |
| *tetO-ARC19/arc19Δ* |  | See Reference | [2] |
| *tetO-ARC40/arc40Δ* |  | See Reference | [2] |
| *tetO-ARC35/arc35Δ* |  | See Reference | [2] |
| *arc40* *Δ/arc40Δ* | CaLC4380 | *arc40:his3::hisG/his3::hisG leu2::tetRGAL4AD-URA3/LEU2 arc40::FRT* | This study. |
| *tetO-RHO1/RHO1* *arc40Δ/Δ* | CaLC4381 | *arc40:his3::hisG/his3::hisG leu2::tetRGAL4AD-URA3/LEU2 arc40::FRT tetO-RHO1/RHO1* | This study |
| *tetO-RHO1/rho1Δ* | CaLC3935 | *arg4/arg4 his1/his1 URA3/ura3::imm434 IRO1/iro1::imm434 tetO::FRT-RHO1/rho1::FRT* | [3] |
| *tetO-RHO1/RHO1^Q67L^* | CaLC4501 | *arg4/arg4 his1/his1 URA3/ura3::imm434 IRO1/iro1::imm434 tetO::FRT-RHO1/RHO1^Q67L^::FRT* | [3] |
| *tetO-RHO1/RHO^T23N^* | CaLC4502 | *arg4/arg4 his1/his1 URA3/ura3::imm434 IRO1/iro1::imm434 tetO::FRT-RHO1/RHO1^T23N^::FRT* | This study |
| *lrg1Δ/ lrg1Δ* | CaLC4090 | As SN95, *lrg1::FRT/lrg1::FRT* | [3] |
| *lrg1Δ/ lrg1Δ PKC1/ pkc1Δ* | CaLC4375 | As SN95, SN95 *lrg1::FRT/lrg1::FRT PKC1/pkc1∷FRT* | [3] |
