# Supplementary material for: Functional Genomic Analysis of Candida albicans Adherence Reveals a Key Role for the Arp2/3 Complex in Cell Wall Remodelling and Biofilm Formation
Source: PLoS Genet. 2016 Nov 21;12(11):e1006452. doi: 10.1371/journal.pgen.1006452 (PMC5147769; doi:10.1371/journal.pgen.1006452)
Supplement: S3 Table — (DOCX) [file pgen.1006452.s003.docx]

Table S3. Plasmids used in this study.

| Plasmid name | Description | Reference |
| --- | --- | --- |
| pLC49 | *FLP-CaNAT, ampR* | [4] |
| pLC605 | *CaTAr-FLP-CaNAT, ampR* | [5] |
| pLC771 | pLC49 *CaRHO1^T23N^*, *ampR*, *NAT* | This study |
| pLC772 | pLC49 *CaRHO1^Q67L^*, *ampR*, *NAT* | [3] |
