# Supplementary material for: Functional Genomic Analysis of Candida albicans Adherence Reveals a Key Role for the Arp2/3 Complex in Cell Wall Remodelling and Biofilm Formation
Source: PLoS Genet. 2016 Nov 21;12(11):e1006452. doi: 10.1371/journal.pgen.1006452 (PMC5147769; doi:10.1371/journal.pgen.1006452)
Supplement: S1 Text — (DOCX) [file pgen.1006452.s005.docx]

**Supplemental Methods**

**Strain Construction**

**CaLC4380**: To construct the *arc40Δ/Δ* strain, the NAT flipper cassette was PCR amplified from pLC49 with oLC4128 and oLC4129 and transformed into CaLC239. NAT-resistant transformants were PCR tested using oLC275 and oLC4131 (1143bp) for upstream integration and oLC274 and oLC4130 (516bp) for downstream integration. The *SAP2* promoter was induced to drive expression of FLP recombinase to excise the NAT flipper cassette. To delete the second allele of *ARC40,* the NAT flipper cassette was PCR amplified from pLC49 with oLC4128 and oLC4129. NAT-resistant transformants were PCR tested using oLC275 and oLC4131 (1143bp) for upstream integration and oLC274 and oLC4130 (516bp) for downstream integration. The *SAP2* promoter was induced to drive expression of FLP recombinase to excise the NAT flipper cassette. The absence of a wild-type allele was verified by PCR with internal primers oLC4177 and oLC4178; the absence of a 382 bp band verified the homozygous deletion.

**CaLC4381:** To regulate the expression of *RHO1* the tetracycline-repressible transactivator, the *tetO* promoter, and the NAT flipper cassette were PCR amplified from pLC605 using primers oLC2838 and oLC2860 and transformed into CaLC4380. NAT-resistant transformants were PCR tested with oLC2862 and oLC534 (522 bp band) and oLC274 and oLC2842 (1223 bp band). The *SAP2* promoter was induced to drive expression of FLP recombinase to excise the NAT flipper cassette.

**CaLC4403:** To regulate the expression of *RHO1* the tetracycline-repressible transactivator, the *tetO* promoter, and the NAT flipper cassette were PCR amplified from pLC605 using primers oLC2838 and oLC2860 and transformed into CaLC3365 (CaSS1: wild type). NAT-resistant transformants were PCR tested with oLC2862 and oLC534 (522 bp band) and oLC274 and oLC2842 (1223 bp band). The *SAP2* promoter was induced to drive expression of FLP recombinase to excise the NAT flipper cassette.

**CaLC4502**: To introduce the T23N mutation into *RHO1*, the *RHO1* complementation vector carrying the T23N mutation was released from pLC771 with BssHII and transformed into CaLC3935. NAT-resistant transformants were PCR tested with oLC275 + oLC2841 (1076 bp) for upstream integration and oLC274 + oLC2868 (483 bp) for downstream integration. The presence of WT *RHO1* promoter was verified by PCR with primers oLC2864 + oLC2843 (1159 bp for WT promoter; 562 bp for the absence of WT promoter) and the presence of a *tetO-RHO1* allele was verified by PCR with primers oLC2862 + oLC534 (522 bp). The *SAP2* promoter was induced to drive expression of FLP recombinase to excise the NAT flipper cassette.

**Plasmid Construction**

**pLC771**: This is a construct to introduce *CaRHO1* carrying the T23N mutation in *C. albicans*. This is based on pLC765 but harbors a mutation in *RHO1* (T23N). This mutation was introduced by site-directed mutagenesis with primers oLC2994 and oLC2995. The clone was sequence verified with oLC2864. The reconstitution construct can be liberated by digestion with BssHII.

**High Throughput Sequencing Library Preparation And Data Analysis**

Amplification of UP and DOWN barcodes by PCR was performed using 78 ng of genomic DNA for GRACE pool samples with the Takara Ex-Taq enzyme (Clontech #RR001) using the following thermal cycler program:

1 94°C, 120s

2 94°C, 2s

3 53°C, 20s

4 72°C, 14s

5 GoTo step 2, × 28

6 72°C, 1min

7 4°C

Separate UPTAG and DNTAG multiplexed pools were formed by combining equal amounts of PCR product from each sample. UPTAG and DNTAG DNA pools were electrophoresed on a 5% 1 × TBE polyacrylamide gel and recovered by eluting DNA from shredded gel slices in 10 mM Tris-HCl pH 8.0. Equal quantities of UPTAG and DNTAG pools were combined to form a library, which was sequenced on an Illumina Hi-Seq 2500 instrument (single-end flow cell) using specific primers to sequence and index the UPTAGs and DNTAGs. Barcode sequence reads were mapped to an artificial genome containing known UPTAG and DNTAG sequences via Bowtie v1. Read frequency for the UPTAG and DNTAG of each strain were compiled for each indexed sample.

Strains where UPTAG and DNTAG read counts were <1 read per million mapped reads were omitted from further analysis. Relative strain abundance in a fraction was calculated by averaging the log2 read counts for the UPTAG and DNTAG. If either barcode in a given strain had <1 read per million mapped reads, only the reads for the complementary barcode in a strain were used to calculate relative strain abundance.

**Supplemental References:**

1. Noble SM, Johnson AD (2005) Strains and strategies for large-scale gene deletion studies of the diploid human fungal pathogen Candida albicans. Eukaryot Cell 4: 298-309.

2. Roemer T, Jiang B, Davison J, Ketela T, Veillette K, et al. (2003) Large-scale essential gene identification in Candida albicans and applications to antifungal drug discovery. Mol Microbiol 50: 167-181.

3. Xie JL, Grahl N, Sless T, Leach MD, Kim SH, et al. (2016) Signaling through Lrg1, Rho1 and Pkc1 Governs *Candida albicans* morphogenesis in response to diverse cues. PLoS Genetics In Press.

4. Morschhauser J, Michel S, Staib P (1999) Sequential gene disruption in Candida albicans by FLP-mediated site-specific recombination. Mol Microbiol 32: 547-556.

5. Leach MD, Cowen LE (2014) Membrane fluidity and temperature sensing are coupled via circuitry comprised of Ole1, Rsp5, and Hsf1 in Candida albicans. Eukaryot Cell 13: 1077-1084.
