# Supplementary figures and images for: Functional Genomic Analysis of Candida albicans Adherence Reveals a Key Role for the Arp2/3 Complex in Cell Wall Remodelling and Biofilm Formation
Source: PLoS Genet. 2016 Nov 21;12(11):e1006452. doi: 10.1371/journal.pgen.1006452 (PMC5147769; doi:10.1371/journal.pgen.1006452)

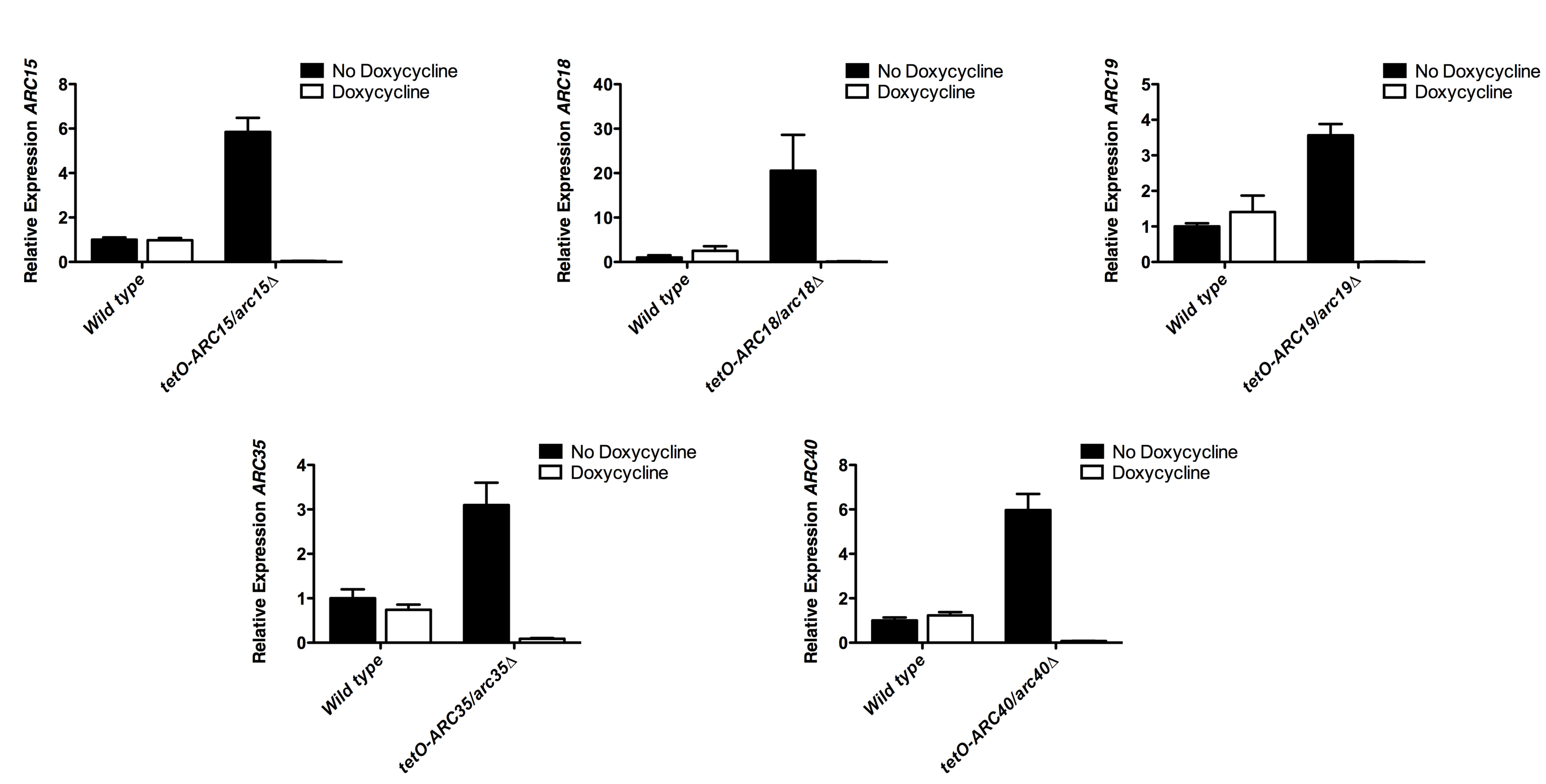

Supplement: S1 Fig — Transcriptional repression of members of the Arp2/3 complex was achieved with 0.5 μg/ml doxycycline. Overnights were subcultured for 24 hrs in the presence or absence of doxycycline. The strains were subcultured again in the same conditions for 4 hrs. cDNA was prepared from total RNA for qRT-PCR. The transcript level of ARC15, ARC18, ARC19, ARC35 and ARC40 was monitored and normalized to GPD1. Data are plotted as means ± SD for triplicate samples and are representative of two independent experiments. (TIF) [file pgen.1006452.s006.tif]

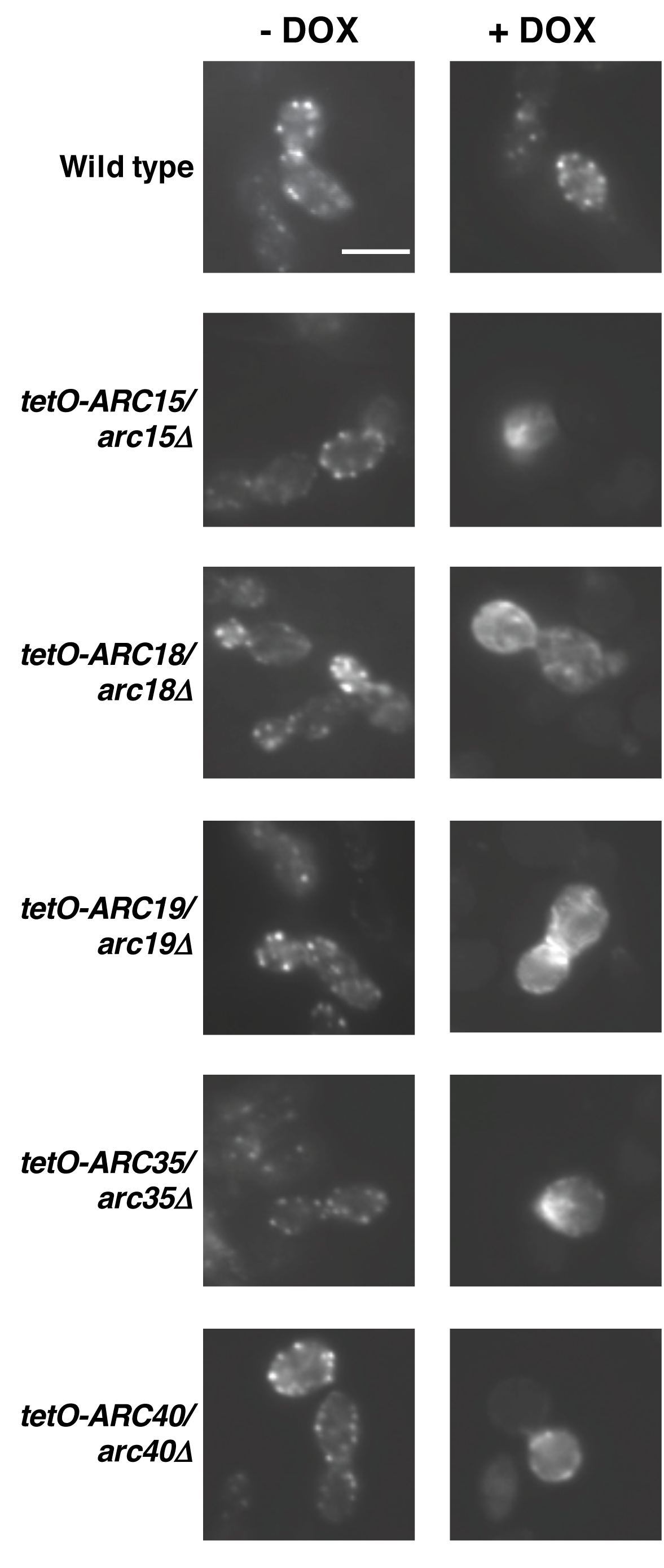

Supplement: S2 Fig — Microscopy images of Arp2/3 complex mutants grown in the absence or presence of doxycycline, and then stained with Rhodamine-Phalloidin. Scale bar represents 10 μm. (TIF) [file pgen.1006452.s007.tif]

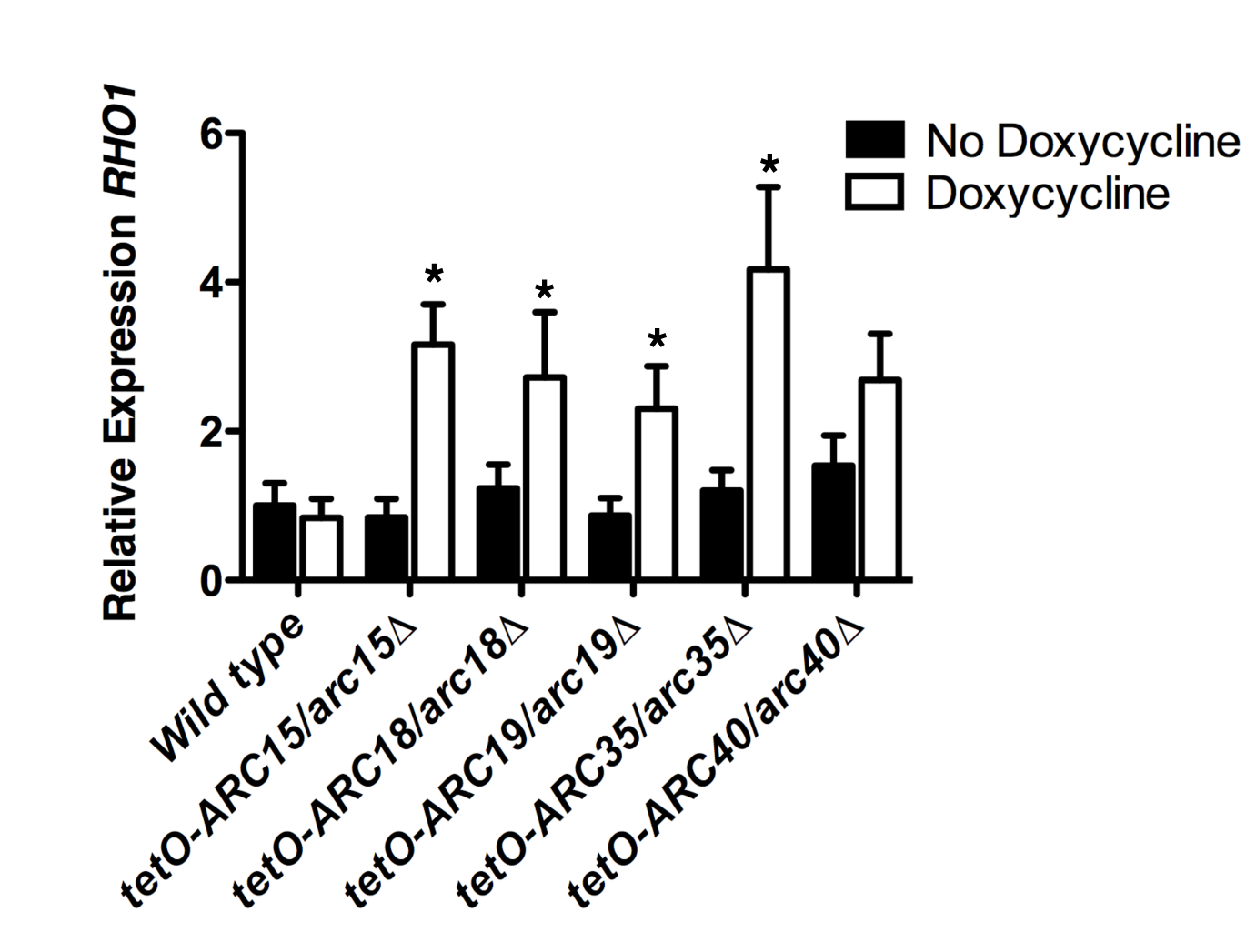

Supplement: S3 Fig — Overnight cultures were subcultured for 24 hrs in the presence or absence of 0.5 μg/ml doxycycline. The strains were subcultured again in the same conditions for 4 hrs. cDNA was prepared from total RNA for qRT-PCR. The transcript level of RHO1 was monitored and normalized to GPD1. Data are plotted as means ± SD for triplicate samples and are representative of two independent experiments. Asterisks indicate significant differences in RHO1 transcript level upon treatment with doxycycline (P<0.05, ANOVA, Bonferroni's Multiple Comparison Test). (TIF) [file pgen.1006452.s008.tif]
